# Induced Abortion: Risk Factors for Adolescent Female Students, a Brazilian Study

**DOI:** 10.1100/tsw.2009.155

**Published:** 2009-12-16

**Authors:** Divanise S. Correia, Jairo C. Cavalcante, Eulàlia M. C. Maia

**Affiliations:** ^1^ Universidade Federal de Alagoas (UFAL), Faculdade de Medicina - Campus A. C. Simões, Cidade Universitària, CEP 57072-900,Maceió AL, Brazil; ^2^ Universidade Federal do Rio Grande do Norte (UFRN), Centro de Ciências da Saúde (CCS), CEP 59072-970 Natal - RN, Brazil

**Keywords:** abortion, risk, adolescence

## Abstract

The purpose of this study was to analyze risk factors for abortion among female teenagers from 12 to 19 years of age in the city of Maceió, Brazil. This is a cross-sectional study, conducted in ten schools. The sample was calculated by considering the number of admissions for postabortion curettage, obtained from the Information System of Hospitalization. Data were obtained through a semi-structured questionnaire divided into three basic blocks of data: sociodemographic, sexual life, and pregnancy/abortion. To analyze the data, the logistic regression model was used. The Forward Method was chosen to set the final model that minimizes the number of variables and maximizes the accuracy of the model. The significant analysis between the dichotomous variables provided eight significant variables. Two of them are protective for abortion: the ages 12-14 years and talking with parents about sex. After the logistic regression, the receipt of support for abortion was the most significant variable of all. The adolescent with an active sexual life, a previous pregnancy, who is married, and has received support for an abortion has a 99.74% probability for an abortion. The results of this study, demonstrating the importance of the group in adolescence, and the statistical significance of having a partner to support and approve the pregnancy appears as a preventive factor for abortion. It shows the importance of support and companionship for adolescent women.

## INTRODUCTION

Abortion, whether induced or not, is an indicator of unplanned pregnancy. Exact information on the occurrence of induced abortion is difficult to obtain, especially in places where it is illegal, as is the case in Brazil[1,2], and where it is a topic of a great sensitivity, involving ethical and moral issues. The total abortion rates are similar in developed and developing countries. Unsafe abortion, however, is concentrated in places where socioeconomic conditions are precarious[1,2,3,4,5,6].

The World Health Organization (WHO) defines unsafe abortion as a procedure performed to terminate an unwanted pregnancy, usually performed by individuals without the necessary skills or in an environment that is not in accordance with required medical standards, or both[7]. It is estimated, worldwide, that in 2003, there were 42 million abortions performed on women between the ages of 15 and 44 years. This represents a decrease from the estimate of 46 million abortions in 1995. On the other hand, in developing countries, the decrease is estimated to have been somewhat smaller, from 3.5 to 3 million[1].

A safe abortion is one that is conducted in accordance with medical standards, with the necessary hygienic care, and where there is a concern for the health of the woman. This type of abortion occurs in countries where there is no legal restriction on abortions and where there are legal restrictions, in legal situations, such as rape or risk of death to the mother. Studies show that in 2003, almost half of the abortions performed were done in unsafe conditions, primarily in less-developed countries. Conversely, in developed countries, 92% of the abortions were safely performed[1,3,6].

In Brazil, abortion is a public health problem. Although prohibited by law, it is widely practiced. Women from less-favored social classes do it in an unsafe way, with unqualified practitioners. Mortality statistics and data related to hospital admissions suggest that abortion is one of the main causes of death in the country[2,7,8,9,10].

The beginning of a sexual life before 15 years of age exposes the female teenager to early unplanned pregnancy and early abortion. Abortion in the early ages of adolescence increasingly appears to be a public health problem[9,10,11].

Sexual changes are a central point in adolescence due to the modifications that they promote in the lives of young people. When young people are able to exercise their sexuality physically, they are still psychologically immature to assume the responsibilities that arise from it[9,11]. In recent years, in Brazil, the increased incidence of pregnancy and abortion between the ages of 10 and 14 has begun to draw attention from public health authorities[10,11,12,13,14]. At this age, teenagers are still in the learning phase of exercising or practicing their sexuality. Adolescents sometimes consider abortion from the moment of discovering an unplanned pregnancy. However, due to its illegality in Brazil, the circumstances through which the youngster makes the choice for abortion, and how to carry it out, are not well known[9,10,11].

It is understood that any analysis on the subject reported here must consider the multiplicity of factors involved at the beginning of a girl’s sexual life, and the issue of gender is a relevant factor. This study works with a representative sample of female teenagers in order to analyze the risk factors provoking abortion in the city of Maceió.

## METHOD

This is an analytical cross-sectional study conducted in ten schools in the city of Maceio in 2005. In this study, the representative and randomized sample was calculated by taking into consideration the number of hospital admissions for curettage (D&C), according to the suggestion provided by Rossier[13,15]. Because abortion is an illegal practice in Brazil, there are no official statistics about it. It is therefore necessary to use other statistical methods.

In Maceió in 2004, the adolescent population between 10 and 19 years of age included 344,221 girls. There were 1,327 hospital admissions for female teenagers for curettage, from a total of 5,622 for all ages. To calculate the sample for this research, and in accordance with the approach of the authors listed above, a figure of 12% of 5,622 was calculated. This value refers indeed to the cases that did not need hospital admission, a total of 675 procedures, from which 25% was deducted. This number refers to 169 spontaneous abortions. The result of deducting 169 from 675 is 506, a value that was multiplied by a

Correction Index of 5, suggested by Correa and Freitas[14], for investigations about abortion in Brazil, which resulted in a minimum sample of 2,530.

This study was undertaken in the Public and Private School System in Maceió and included those schools that have Junior and High School levels, the age group used in the study. Then, the number to be found in each school was calculated and it was evident that ten schools would be enough for the attainment of the established sample. From this, the drawing of the educational institutions was carried through, considering 50% for public and 50% for private.

This research is part of a larger study about abortion. Data were obtained through a semi-structured questionnaire divided into three basic blocks of data: sociodemographic, sexual life, and pregnancy/abortion.

The questionnaire comprised 53 questions, developed after a literature review, and was applied by five pairs of medical and psychology students who were previously trained.

The choice of university students was an intent to provide interlocutors closer in age to the subjects and made use of the knowledge they acquired during their course of studies, allowing a more open dialogue on the subject. The questionnaire was administered in the classroom, at a set time, when Term of Free and Informed Consent had been obtained from the participants and their parents or guardians. A pilot study was conducted to test the questionnaire.

To analyze the data, the logistic regression model was used, together with the stepwise technique based on the statistical criteria of the Wald test. The Forward Method was chosen to set the final model that minimizes the number of variables and maximizes the accuracy of the model[17].

The dependent variable was the practice of abortion and the independents' age, marital status, sexual life, previous pregnancy, use of contraceptive method, talk with parents about sex, support for abortion, and need of hospitalization postabortion.

At first, there was an analysis of dichotomous variables using the program Epi Info (Version 3.3.2) and the values of the odds ratio with confidence interval 95%. Then, using the program SPSS 13.0, a logistic regression was calculated that included the significant variables.

The selection of the dichotomous independent variables showed significant association with the dependent variable (the practice of abortion).

The logistic regression is a transformation of the binary answer in linear form that can be described for:

$$\ln(\text{ODDS}) = \beta_0 + (\beta_1 * X_1) + (\beta_2 * X_2) + \ldots + (\beta i * Xi)$$

$$P = e^{\ln(\text{ODDS})}/(1 + e^{\ln(\text{ODDS})})$$

$$e = 2.718281828$$

The parameter $\beta$ has meaning similar to the linear regression. The ln (odds) is also called the Logit Function and it has a lot of properties of the linear model[18].

## RESULTS

Of the 2,592 teens surveyed, most were between 15 and 19 years old (70.1%) and single (95.7%). They did not talk with their parents about sex (74.4%) and were not sexually active (78.4%). Rates of 7 and 5.7% of pregnancies and abortion, respectively, were found (Table 1).

The sexually active young people presented the same general distribution of the group, i.e., most were over 15 (90.3%), were single (85.3%), had not been previously pregnancy (67.6%) or had an abortion (73.3%). Those who had abortions, however, received support to do that (63.8%) (Table 2).

**TABLE 1**
**Characteristics of Adolescents Surveyed**

| Characteristics | Age (Years) | | | | Total | |
|---|---|---|---|---|---|---|
| | 12–14 | | 15–19 | | n = 2,592 | |
| | n = 775 | 29.9% | n = 1,817 | 70.1% | n | % |
| Marital status | | | | | | |
| Single | 762 | 30.7 | 1,719 | 69.3 | 2,481 | 95.7 |
| Married | 13 | 11.7 | 98 | 88.3 | 111 | 4.3 |
| Talk about sex with parents | | | | | | |
| Yes | 180 | 27.1 | 484 | 72.9 | 664 | 25.6 |
| No | 595 | 30.9 | 1333 | 69.1 | 1,928 | 74.4 |
| Sexual life | | | | | | |
| Yes | 54 | 9.7 | 505 | 90.3 | 559 | 21.6 |
| No | 721 | 35.5 | 1,312 | 64.5 | 2,033 | 78.4 |
| Use of contraceptive methods | | | | | | |
| Yes | 24 | 6.8 | 328 | 93.2 | 352 | 13.6 |
| No | 751 | 33.5 | 1,489 | 66.5 | 2,240 | 86.4 |
| Pregnancy | | | | | | |
| Yes | 13 | 7.2 | 168 | 92.8 | 181 | 7.0 |
| No | 762 | 31.6 | 1,649 | 68.4 | 2,411 | 93.0 |
| Abortion | | | | | | |
| Yes | 11 | 8.7 | 138 | 91.3 | 149 | 5.7 |
| No | 762 | 31.2 | 1,681 | 68.8 | 2,443 | 94.3 |

**TABLE 2**
**Characterization of Adolescents with Sexually Active Life**

| Characteristics | Age (Years) | | | | Total | |
|---|---|---|---|---|---|---|
| | 12–14 | | 15–19 | | n = 559 | |
| | n = 54 | 9.7% | n = 505 | 90.3% | n | % |
| Marital status | | | | | | |
| Single | 52 | 10.9 | 425 | 89.1 | 477 | 85.3 |
| Married | 2 | 2.4 | 80 | 97.6 | 82 | 14.7 |
| Previous pregnancy | | | | | | |
| Yes | 13 | 7.2 | 168 | 92.8 | 181 | 32.5 |
| No | 41 | 10.8 | 337 | 89.2 | 378 | 67.6 |
| Abortion | | | | | | |
| Yes | 11 | 7.4 | 138 | 92.6 | 149 | 26.7 |
| No | 43 | 10.5 | 367 | 89.5 | 410 | 73.3 |
| Support for aborting | | | | | | |
| Yes | 5 | 5.3 | 90 | 94.7 | 95 | 63.8 |
| No | 6 | 11.3 | 48 | 90.7 | 54 | 36.2 |

The significant analysis between the dichotomous variables, being the variable abortion, and the other independent variables using odds ratio test, provided eight significant variables. Two of them are protective for abortion: the ages 12–14 (Odds = 0.21, $CI_{95\%}$ = 0.19–0.37) and talking with parents about sex (Odds = 0.63, $CI_{95\%}$ = 0.41–0.96). The other independent variables (marital status with a partner, sexually active life, previous pregnancy, use of contraceptive method, support for abortion, and need for hospital postabortion) provided are statically significant, encouraging abortion by presenting Odds greater than 1 (Table 3).

**TABLE 3**
**Dichotomous Independent Variables Worked**

| Independent Variable | Odds Ratio | $CI_{95\%}$ |
|---|---|---|
| Age 12–14 | 0.21 | 0.19–0.37 |
| Marital status: married | 4.58 | 2.78–7.55 |
| Sexual life | 177.66 | 65.42–482.46 |
| Previous pregnancy | 1,872.78 | 725.07–4,837.16 |
| Use of contraceptive method | 13.55 | 9.51–19.32 |
| Talk with parents about sex | 0.63 | 0.41–0.96 |
| Support for abortion received | 1,712.64 | 412.21–7,115.57 |
| Need for hospitalization postabortion | 273.36 | 35.84–2,084.99 |

The regression was performed in four steps. In the first step, the variable was the previous pregnancy (Odds = 1,872.78, $CI_{95\%}$ = 725.09–4,837.08); in the second step, the variable was the support for abortion (Odds = 869.56, $CI_{95\%}$ = 98.02–7,713.87); in the third step, the variable was marital status with a partner, which has a protective value (Odds = 0.26, $CI_{95\%}$ = 0.10–0.69); and the fourth and last step was the sexual life variable ( Odds = 8.20, $CI_{95\%}$ = 1.88–35.76) (Table 4).

**TABLE 4**
**Steps Taken in Logistic Regression**

| Step | Variables | B | S.E. | Wald | Sig. | Odds Ratio | $IC_{95\%}$ Lower | $IC_{95\%}$ Upper |
|---|---|---|---|---|---|---|---|---|
| 1 | Previous pregnancy | 7.535 | 0.48 | 242.24 | 0.000 | 1,872.778 | 725.086 | 4,837.079 |
| | Constant | –6.176 | 0.45 | 190.34 | 0.000 | 0.002 | | |
| 2 | Previous pregnancy | 7.176 | 0.61 | 136.83 | 0.000 | 1,307.907 | 392.994 | 4,352.797 |
| | Support for abortion received | 6.768 | 1.11 | 36.93 | 0.000 | 869.561 | 98.023 | 7,713.870 |
| | Constant | –6.707 | 0.58 | 134.62 | 0.000 | 0.001 | | |
| 3 | Previous pregnancy | 7.509 | 0.63 | 142.08 | 0.000 | 1,825.110 | 530.920 | 6,274.062 |
| | Marital status: married | –1.336 | 0.49 | 7.40 | 0.007 | 0.263 | 0.100 | 0.688 |
| | Support for abortion received | 6.747 | 1.11 | 36.76 | 0.000 | 851.374 | 96.126 | 7,540.474 |
| | Constant | –6.684 | 0.58 | 133.70 | 0.000 | 0.001 | | |
| 4 | Sexual life | 2.104 | 0.75 | 7.84 | 0.005 | 8.198 | 1.879 | 35.764 |
| | Previous pregnancy | 6.298 | 0.69 | 84.23 | 0.000 | 543.743 | 141.657 | 2,087.121 |
| | Marital status: married | –1.505 | 0.50 | 9.18 | 0.002 | 0.222 | 0.084 | 0.588 |
| | Support for abortion received | 6.463 | 1.21 | 28.55 | 0.000 | 641.100 | 59.891 | 6,862.573 |
| | Constant | –7.411 | 0.73 | 104.38 | 0.000 | 0.001 | | |

After the logistic regression, there remained only four significant variables in the model from the original eight, with adjusted Odds. The receipt of support for abortion was the most significant variable of all (Odds = 641.10, $CI_{95\%}$ = 59.89–6,862.57) to provoke abortion, and marital status with a partner was a protective factor (Odds = 0.22, $CI_{95\%}$ = 0.08–0.59).

Based on the results of the step 4, we built the following prediction model (Table 4):

$$Ln(odds) = Ln(P/(1-P)) = –7.411 + (2.104*Sexual life) + (6.298*Previous Pregnancy) + (–1.505*Marital status: married) + (6.463*Support for abortion received)$$

$$Ln(odds) = Ln(P/(1-P)) = –7.411 + (2.104*1) + (6.298*1) + (–1.505*1) + (6.463*1) = 5.949$$

$$P = e^{5.949}/(1+e^{5.949}) = 0.9974\ (99.74\%)$$

A youth with an active sexual life, a previous pregnancy, who is married, and has received support for an abortion has a probability of abortion of 0.9974 (99.74%).

## DISCUSSION

In Brazil, the beginning of sexual activity before the national average age of 15 is associated with various socioeconomic factors. Social factors associated with premature pregnancy include lack of information and a reduced use of contraceptive methods, together with the lack of education through health services and school about adolescent sexual development[19,20,21,22,23].

Sexuality is one of the things that undergoes the most changes during the period of adolescence. The best way to prevent the risks that the exercise of sexuality in this age group brings about is to talk about sex within the family. This practice, however, is not easy[9].

The sexual and reproductive behavior of teenagers is not only determined by their individual characteristics, it is also determined by the social context in which the adolescents are found. Community characteristics do affect the youngsters, creating situations that shape their knowledge and attitudes, consequently directing their choices in relation to their sexual behavior[9,21,23].

It was found in this study that only 25.6% of respondents talked with their parents about sex (Table 1), and subsequent information from the girls showed that this dialogue usually happens only with the mother. The literature shows that when a girl has an open dialogue with her mother from her childhood about sexuality, menarche, menstruation, and sex, she starts her sexual life much later than other girls who have not had this opportunity[4,8,12,19].

The literature also refers to the contribution of economic instability to early sexual activity, including school evasion, the low levels of education, and a limited access to work opportunities, particularly when family support is not available[9]. Adolescents in Brazil belong in a world where dialogue about sexuality is not a common practice. The adolescent is the result of his/her internal family traditions and of the symbolical instructions received from discussion within the home, as well as community beliefs and dogmas[22]

For girls who do not talk about sex with a parent, their closest friend becomes important in a sexual crisis and takes up part of the role not filled by the parents[19]. This demonstrates the need for educational and preventive activities among girls for the issues studied here.

It is observed that of 559 adolescents who had sex and became pregnant (32.5%), the majority claimed to have had abortions (26.7%) (Table 2). An important reflection on this issue that is not addressed directly by this study is: Why are the teenagers actually becoming pregnant and aborting in such a rate? Was it the case that those who opted to maintain their pregnancy left school and did not appear in this statistical research, which was based in the school environment?

Table 2 also shows the percent of pregnant teenagers who received support (63.8) to abort. This shows once again the malfunction of the youngsters' preventive and health assistance system, as the support came mainly from female friends (32.9%) and adolescent peers (25.5%). It should also be noted that this is related to the illegality of the act and, therefore, the insecurity of its practice in Brazil.

Abortion in illegal conditions usually occurs in a situation where a woman is exposed to risks such as septic abortion. This is more common among adolescents because of the characteristics of their physical development. Adolescents are significantly at risk of septic abortion because of their age and because they take longer to admit and recognize that they are pregnant. Risks of abortion are lower in countries where abortion is legal and among older women who know where to look for help[3].

Of the eight dichotomous variables studied here (Table 3), significant when studied before, only four were found to be significant (Table 4) and the support for abortion was the most significant variable in this study when performing the regression.

Having a stable partner appears to be a protective factor, corroborating studies on abortion in adolescence that show the importance of the partners' support in the continuation of pregnancy[8,9,10,20]. This demonstrates the necessity for further studies about this subject with the participation of male adolescents.

In spite of the fact that abortion has been a constant theme in the scientific literature in Brazil, increasing in the mid-20th century, there is no research on abortion in the North, while 14% of the studies were conducted in the Northeast and 4% in the Central-West.

The trustworthy statistics on provoked abortion from countries where this practice is generalized allow for an adequate evaluation of the impact of abortion on reproductive health. Paradoxically, it is in these very countries that the less-severe damage to reproductive health is observed. At the same time, even based on little or totally untrustworthy data, the cost of this clandestine practice is very high; its sequels are frequent and often lead to death.

The authors recognize the limitations of the study. However, the results can be important when studying the abortion issue in places with similar economic situations as Brazil and where the abortion practice is illegal.

## CONCLUSION

Support for abortion was found to be the most significant variable in this study, demonstrating once again the importance of the group in adolescence and the need to consider education as a preparation and a preventive action for the reproductive health of young people.

The statistical significance of having a partner to support and approve the pregnancy appears to be a preventive factor for the practice of abortion. A youth with an active sexual life, a previous pregnancy, who is married, and has received support for an abortion has a probability of abortion of 0.9974 (99.74%). It shows the importance of support and companionship for adolescent women.

Public policy makers are urged to reflect and act on the data in this study that can provide information and assistance to prevent abortions in Maceió.
